# Depression among married individuals: new evidence in Türkiye

**DOI:** 10.3389/fpsyt.2026.1749926

**Published:** 2026-05-11

**Authors:** Şeyda Ünver, Ömer Alkan, Ensar Ağırman

**Affiliations:** 1 Department of Econometrics, Faculty of Economics and Administrative Sciences, Ataturk University, Erzurum, Türkiye; 2 Master Araştırma Eğitim ve Danışmanlık Hizmetleri Ltd. Şti., Erzurum, Türkiye; 3 Department of Business Administration, Faculty of Economics and Administrative Sciences, Ataturk University, Erzurum, Türkiye

**Keywords:** binary logistic regression, depression, married individuals, Türkiye, Türkiye Health Survey

## Abstract

**Introduction:**

Common mental disorders (CMDs) are frequently referred to as the “hidden epidemic” of the twenty-first century, even though mental health is widely acknowledged as a fundamental human right on a global scale. Mood, cognition, and behaviour disorders that impact a person’s physical, psychological, and social well-being define CMDs. In addition to directly harming individuals, CMDs have a significant influence on families, society, and the country as a whole. This study focuses exclusively on married individuals and aims to examine how their experiences of depression are associated with demographic and socioeconomic characteristics.

**Methods:**

The Turkish Statistical Institute’s (TurkStat) 2022 Türkiye Health Survey microdata set, which included information on 15,049 married people aged 15 and above, was used in this study. The chi-square test of independence was used to investigate the connection between the independent factors and the experience of depression. Binary logistic regression analysis was used to determine the elements influencing married people’s experiences with depression.

**Results:**

According to the results of the binary logistic regression analysis, it was determined that the variables of gender, age, education level, general health status, employment status, disease, alcohol use, tobacco use, social security status, history of heart attack, and experience of diabetes were associated with the experience of depression.

**Discussion:**

Marriage and family bonds are essential and vital social frameworks for every society. The family is a crucial social setting for children’s, teens’, and adults’ health. Therefore, improving the family environment will be beneficial not only for individuals but also for the entire society. According to the study, social policies are needed to improve the family environment. Psychosocial counselling should be provided to individuals starting from the pre-marital period. Family therapy and counselling services should be more accessible for married individuals.

## 1 Introduction

Common mental illnesses (CMDs) are frequently referred to as the “silent epidemic” of the twenty-first century, even though mental health is widely acknowledged as a fundamental human right (1). In addition to directly harming individuals, CMDs have significant repercussions for families, society, and the country as a whole (2). One of the most prevalent CMDs is depression (3).

A persistent decline in emotional well-being characterizes depression. It affects 5 per cent of the global population and contributes to the worldwide burden of disease and disability (4). Depression affects not only individuals but also their families and overall quality of life; depressive episodes are associated with organic problems such as chronic back pain and migraine attacks (5, 6), as well as behavioral problems such as insomnia and anger (7, 8), and may disrupt family interactions (9). Depression is also associated with profound emotional distress, an increased risk of somatic conditions such as coronary heart disease, and the global burden of disability and suicide (10). Clinically, depressive episodes are classified as unipolar or bipolar depending on the presence or absence of manic or hypomanic episodes (11). In particular, patients with bipolar disorder treated with antidepressants alone may show poor symptom improvement, a tendency to transition into manic episodes, and an increased risk of suicide, underscoring the importance of accurate early diagnosis (11, 12). (11). Given that depression affects not only individuals but also their interpersonal relationships and family dynamics, it is particularly important to examine how core social institutions, such as marriage, shape mental health outcomes.

One of the most significant and long-lasting interpersonal bonds is marriage. According to Cutler and Radford (13), marriage is a system of people who voluntarily spend time together, speak honestly, feel a sense of duty, support one another socially, and come together in times of need. Marriage is one of the oldest social institutions. Supported by religions, laws, and social norms throughout human history, marriage is an enduring feature in nearly all cultures (14). Given its central role in shaping individuals’ social and relational environments, marriage has also been widely examined in relation to mental health outcomes.

To illustrate how marriage preserves mental health, two opposing but frequently overlapping theories are presented. First, according to the marital protection thesis, married people are surrounded by a better set of social settings and practices than single people (15). The institution of marriage is known to increase access to public acceptance and social support, both of which are linked to better mental health outcomes (16). Second, according to the marital selection argument, married people are a stronger and healthier group than single people (17). Although longitudinal testing of the selection impact hypothesis has been conducted, the results are conflicting (18). Some studies find that mentally and physically healthier individuals are more likely to marry, while others argue that marriage itself strongly affects health and well-being (19). Some contradictory studies suggest that less healthy individuals, especially those at higher risk for depression, may choose marriage and then benefit from its protective health effects (20, 21). In summary, although findings are mixed, research suggests that both mechanisms may be at work: individuals who are mentally more resilient tend to choose marriage, and marriage, in turn, provides protective benefits for mental health (16).

A substantial body of literature addresses the mechanisms through which marriage may influence mental health. For instance, experimental studies have demonstrated that married individuals reporting lower emotional satisfaction (22), distressed spousal relationships (23), poor communication (24), and perceived injustice in household task division report higher depressive symptoms in both United States-based and international studies (16, 25). Additionally, it is well known that major depression is a prevalent and recurrent condition (26, 27).

Previous studies have found that major depressive disorder is present in adult populations (16, 28, 29), and numerous researchers have provided evidence of a significant concurrent negative relationship between marital quality and depressive symptoms (30–33). Likewise, a different study discovered that women had a stronger correlation between marital adjustment and current depressive episodes than did males (28). After adjusting for comorbid conditions, marital dissatisfaction was substantially linked to severe depression in women and dysthymia in males in a prior study that looked at a variety of mental diseases (16). In contrast, a different earlier study discovered that only men had a substantial correlation between marital quality and depressed symptoms (34). Nonetheless, a large amount of data indicates that there is no gender-related variation in the magnitude of the contemporaneous relationship between depression and marital quality (29, 35, 36).

Since the onset and course of depression in married individuals are shaped by the social roles and norms associated with marriage, it is important to examine international studies that reveal how this relationship varies across different sociocultural contexts. Although a link between marital status and mental health has been established, most existing studies examining the relationship between marital status and depression have relied on data from a single country—primarily Western nations—and generally indicate that being married has a protective effect against depression (37, 38). However, these patterns may not be generalizable globally, as other countries differ from Western countries in many ways. Differences in culture, socioeconomic development, and education can uniquely shape marital behaviors across countries (39). For example, studies conducted in Korea and Kenya found no association between marital status and depression among women (40, 41).

Even within Europe, studies show that the psychological consequences associated with widowhood vary widely due to limited sample sizes (42). Therefore, there is a critical need for large-scale, cross-national analyses to clarify these complex relationships. A previous study aimed to examine the relationship between marital status and the risk of developing both current and future depressive symptoms, as well as to identify potential moderating and causal mediating variables (10).

Depression is widely recognized as a multifactorial mental health condition shaped by the interaction of genetic, environmental, and socioeconomic factors (11). A growing body of empirical research has examined the determinants of depression among married individuals across different contexts. Studies highlight the importance of household dynamics, showing that greater spousal participation in domestic work is associated with a lower risk of depressive symptoms among married women (43). Similarly, physical activity has been found to reduce depression risk among married women, although this association may vary by gender (44). Family structure and relational proximity also play a role, with variations in living arrangements and intergenerational co-residence influencing depression levels in complex ways (45). Moreover, family-related factors have been shown to mediate the relationship between marriage or cohabitation and depression (46).

Socioeconomic and contextual factors further shape depression risk. Evidence indicates that lower socioeconomic status, lower education, larger family size, and adverse life events are associated with higher levels of depression among married women (47). Work–family conflict is another important determinant, increasing the likelihood of depressive symptoms among married working women (48). Similarly, higher prevalence rates of depression have been documented among married women in specific regional contexts (49).

At the same time, protective factors have also been identified. Marriage itself may reduce the likelihood of depressive symptoms in certain contexts, such as among individuals in same-sex relationships (50). In addition, individual-level psychosocial characteristics, including self-esteem, are negatively associated with depression (51). Evidence also indicates that the prevalence of anxiety and depression among married women of reproductive age varies across contexts, with reported rates of 19.5% and 4.9%, respectively, in Bangladesh (3). Furthermore, findings from Türkiye suggest that family environment is associated with stress and anxiety levels, with a weak positive relationship between stress and anxiety (r = 0.306) and a weak negative association between family environment and anxiety (52). Overall, the literature demonstrates that depression among married individuals is shaped by a complex interplay of relational, socioeconomic, and individual-level factors, with findings varying across cultural and contextual settings.

Recent high-quality studies have strengthened the evidence on the socioeconomic determinants of depression, showing that structural factors such as education, income, and employment status play a central role in shaping mental health outcomes. These findings suggest that depression should not be viewed solely as an individual-level condition but rather as a phenomenon embedded within broader socioeconomic systems. In this context, examining depression within specific social institutions, such as marriage, provides an important opportunity to better understand how structural and relational factors jointly influence mental health.

Depression is a common condition that affects individuals’ overall functioning and has important consequences not only for psychological well-being but also for family relationships and broader socioeconomic outcomes. Accordingly, this study aims to identify the demographic and socioeconomic determinants of depression among married individuals in Türkiye.

This study makes several important contributions to the literature. First, it provides one of the largest nationally representative analyses focusing on married individuals in Türkiye, thereby offering context-specific evidence from a non-Western setting. Second, by integrating the marital protection and marital selection frameworks, the study contributes to the theoretical debate by demonstrating that these mechanisms may operate simultaneously rather than independently. Third, the findings highlight the role of socioeconomic and health-related factors in shaping depression risk within marriage, emphasizing that mental health outcomes are influenced not only by marital status but also by structural inequalities and institutional conditions. Finally, the study offers policy-relevant insights by identifying high-risk groups and linking empirical findings to context-specific intervention strategies.

## 2 Method

### 2.1 Sample

**Figure 1:**
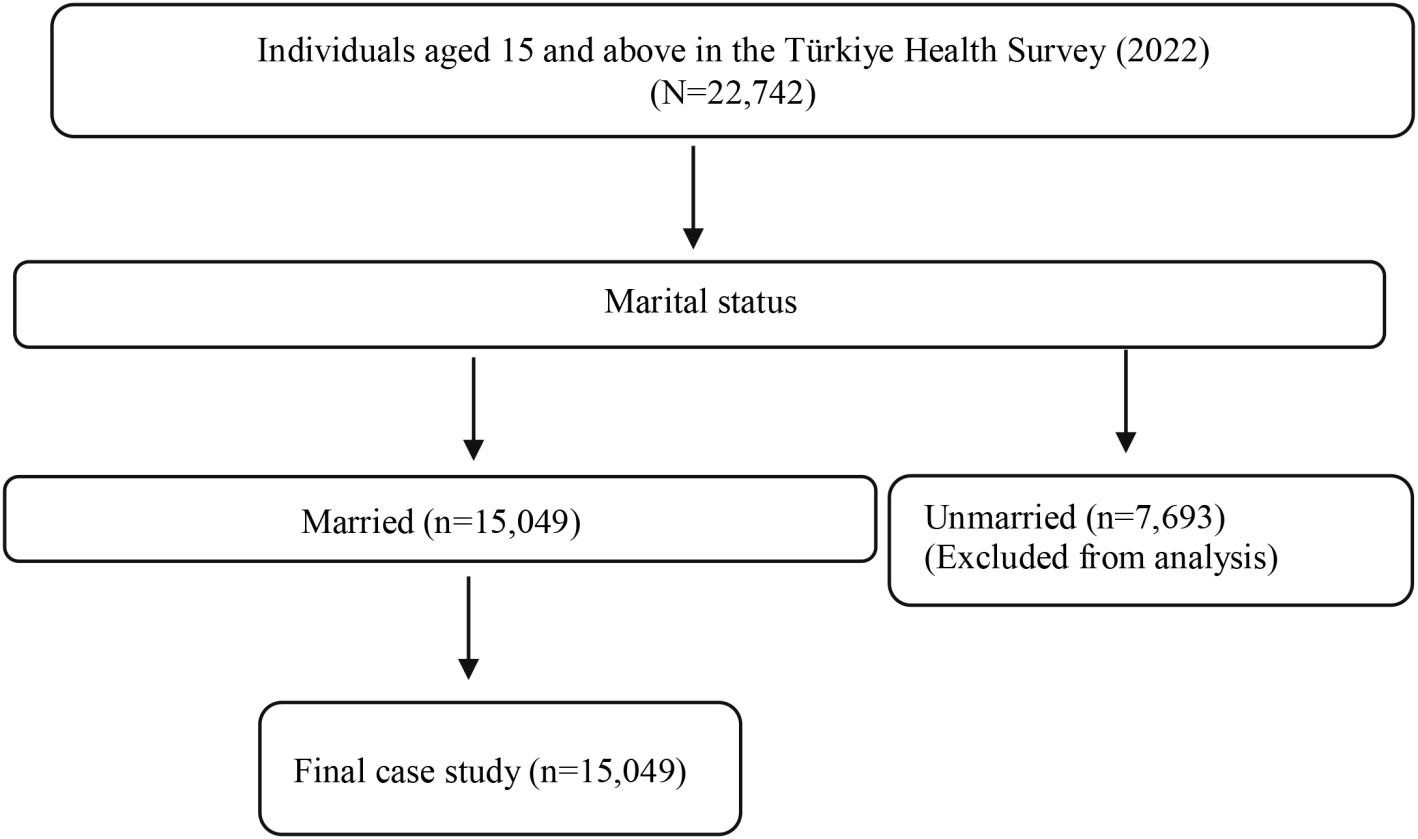
Selection process of married women among individuals in the THS.

The investigation utilized data from the Türkiye Health Survey conducted by the Turkish Statistical Institute (TurkStat) in 2022. Data from 15,049 married individuals who participated in the Türkiye Health Survey were used in this study (53). The selection process of the sample to be included in the study is given in **Figure 1**. Regarding missing data, the dataset used in this study did not contain missing observations for the variables included in the analysis. Therefore, all available observations were retained, and no imputation or case-wise deletion procedures were required. The absence of missing data strengthens the robustness of the findings by eliminating potential biases associated with non-random missingness. Consequently, the estimated results are based on the full sample and are not affected by data incompleteness.

### 2.2 Outcome variables

The dependent variable of the study is the presence of depression experienced by married individuals within the last 12 months. If respondents reported experiencing depression in the previous 12 months at the time of the survey, the variable was coded as “1”; otherwise, it was coded as “0”.

Short scales are increasingly used in epidemiological studies to measure the physical and mental health of populations, thereby reducing the burden on participants, simplifying administration and translation, and providing efficient global health indicators (54). The use of single-item measures is also on the rise (55). This includes the self-rated mental health (SRMH) measure: “In general, how would you describe your mental health: Excellent, Very Good, Good, Fair, or Poor?” The first use of a single SRMH item was in studies conducted in the 1970s with university students regarding personality traits and help-seeking for mental health (56, 57). In 1981, it was used as part of the National Institute of Health Diagnostic Interview Programme, developed using criteria from the Diagnostic and Statistical Manual of Mental Disorders-III (58). A single SRMH item was subsequently included in the World Health Organisation’s International Diagnostic Interview (59). More recently, the SRMH item has been used as an independent indicator of mental health in both small- and large-scale studies (55).

Examples of national epidemiological studies in which the SRMH measure is used independently include the Canadian Community Health Survey and the Panel Study of Medical Expenditure (60, 61). Researchers have used the SRMH to examine mental disorders (62), care needs (63), patterns of use (64) and adherence to treatment plans (20). Others have examined its relationship with valid clinical measures for diagnosing mental health conditions (62, 65). The measurement of depression using a single self-report question is also documented in the current literature (66–70).

### 2.3 Independent variables

The study’s independent variables are those identified by a review of the pertinent literature and those found in the Türkiye Health Survey. The independent variables are: gender (male, female) (71–74), age (15-24, 25-34, 35-44, 45-54, 55-64, 65+) (75, 76), educational status (illiterate, primary school, elementary school, high school, university) (77, 78), employment status (unemployed, employed) (67, 79), general health status (very good/good, moderate, poor/very poor) (80, 81), presence of a long-term illness or health problem that has lasted or is expected to last six months or more (no, yes) (82, 83), experience of a heart attack within the last 12 months (no, yes) (81, 84), diagnosis of diabetes (81), experience of a substance use-related illness in the previous 12 months (no, yes), alcohol use (no, yes) (16, 85), tobacco use (no, yes) (67, 68), whether the Social Security Institution (SSI) covers healthcare expenses (no, yes), and use of e-cigarettes (no, yes) (86, 87).

Ordinal and nominal variables were defined as dummy variables to observe the effects of all categories of variables included in the binary logit model (88, 89).

### 2.4 Statistical analysis

The dataset obtained from the sample was weighted to account for selection probabilities in the multi-stage sample design (90). A weighted analysis used Stata 17 survey statistics to handle the intricate sampling design and weights (91). A chi-square test of independence was used to investigate the association between the independent variables and the prevalence of depression in married people. Binary logistic regression analysis was then used to identify the elements impacting married people’s risk of developing depression.

Non-parametric statistics are used for categorical data (nominal, ordinal). Logistic regression, which is a non-parametric statistical method, is used when the dependent variable is categorical with exactly two outcomes (92).

In social sciences, especially in socio-economic research, some of the variables examined are measured on a sensitive scale, while others consist of dichotomous data such as positive-negative, successful-unsuccessful, and yes-no. Dichotomous data are the most commonly used form of categorical data. When the dependent variable is dichotomous categorical data, logistic regression analysis is used to examine the cause-and-effect relationship between the dependent variable and the independent variable(s) (93).

Logistic regression is a statistical method that allows for classification in accordance with probability rules by calculating the predicted values of the dependent variable as probabilities (94).

The logistic model was initially developed for use in survival analysis. Here, the dependent variable (Y) takes values of 1 or 0, depending on whether the event of interest occurs (95). The expected value, E(Y), never falls below 0 or above 1. Therefore, the predicted values of $\hat{y}$ in the logistic model range between 0 and 1 (96, 97).

Logistic model is written as,

(1) $$E(Y)=\;\pi =P(Y=1)=\frac{e^{\beta_{0}+\;\beta_{1}X_{i}}}{1+\;e^{\beta_{0}+\;\beta_{1}X_{i}}}\;or\;\frac{\text{exp}(\beta_{0}+\beta_{0}X_{i})}{1+\text{exp}(\beta_{0}+\beta_{0}X_{i})}\;\;\;\;\;$$

After dividing the numerator and denominator of Equation 1 by $e^{\beta_{0}+\;\beta_{1}X_{i}}$ or exp $\left({\text{β}}_{0}+{\text{β}}_{0}{\text{X}}_{\text{i}}\right)$,

(2) $$E(Y)=\;\pi =\frac{1}{1+e^{-(\beta_{0}+\;\beta_{1}X_{i})}}\;or\;\frac{1}{1+\text{exp}(-\beta_{0}-\beta_{0}X_{i})}$$

In the equation (Equation 2), there is a condition that $Y=\{\begin{array}{c} 1,\;\;if\;event\;A\;occurs\\ 0,\;\;if\;event\;B\;occurs \end{array}$ and X values are qualitative or quantitative independent variables.

## 3 Results

### 3.1 Descriptive statistics and chi-square tests

Findings related to the factors that may influence the likelihood of depression among married individuals in Türkiye are presented in **Table 1**. Among the married individuals experiencing depression, 31.7% are male. While 43.1% of those experiencing depression are primary school graduates, 13.2% hold a university degree. It is observed that 25.7% of the married individuals who experienced depression in the last 12 months are in the 45–54 age group. Examining **Table 1**, it is found that 11.9% of married individuals who experienced depression in the past 12 months reported alcohol use. In addition, 6.2% of these individuals reported having a heart attack within the same period.

**Table 1:** Findings concerning the variables influencing the likelihood of depression in married individuals.

Variables		Depression status of married individuals		n (%)	χ2	P
		No	Yes			
Gender	Male	7,053(50.2)	318(31.7)	7,371(49.0)	127.727	0.000
	Female	6,994(49.8)	684(68.3)	7,678(51.0)		
Age	15-24	346(2.5)	16(1.6)	362(2.4)	56.967	0.000
	25-34	2,524(18.0)	116(11.6)	2,640(17.5)		
	35-44	3,675(26.2)	222(22.2)	3,897(25.9)		
	45-54	3,116(22.2)	258(25.7)	3,374(22.4)		
	55-64	2,430(17.3)	234(23.4)	2,664(17.7)		
	65+	1,956(13.9)	156(15.6)	2,112(14.0)		
Educational Status	Illiterate	1,406(10.0)	132(13.2)	1,538(10.2)	41.571	0.000
	Primary school	5,248(37.4)	432(43.1)	5,680(37.7)		
	Elementary school	1,869(13.3)	130(13.0)	1,999(13.3)		
	High school	2,703(19.2)	176(17.6)	2,879(19.1)		
	University	2,821(20.1)	132(13.2)	2,953(19.6)		
Employment Status	Unemployed	7,804(55.6)	721(72.0)	8,525(56.6)	102.429	0.000
	Employed	6,243(44.4)	281(28.0)	6,524(43.4)		
Disease Status	No	6,172(43.9)	18(1.8)	6,190(41.1)	685.977	0.000
	Yes	7,875(56.1)	984(98.2)	8,859(58.9)		
General Health Status	Very good/good	8,613(61.3)	238(23.8)	8,851(58.8)	647.563	0.000
	Moderate	4,429(31.5)	532(53.1)	4,961(33.0)		
Poor/very poor	1,005(7.2)	232(23.2)	1,237(8.2)		
Alcohol Use Status	No	12,452(88.6)	883(88.1)	13,335(88.6)	0.252	0.326
	Yes	1,595(11.4)	119(11.9)	1,714(11.4)		
Experiencing Illness due to Substance Use	No	14,045(99.99)	1,001(99.9)	15,046(99.99)	3.435	0.187
	Yes	2(0.01)	1(0.1)	3(0.01)		
Tobacco Use Status	No	9,412(67.0)	628(62.7)	10,040(66.7)	7.893	0.003
	Yes	4,635(33.0)	374(37.3)	5,009(33.3)		
E-cigarette Use Status	No	13,867(98.7)	995(99.3)	14,862(98.8)	2.589	0.064
	Yes	180(1.3)	7(0.7)	187(1.2)		
SSI Coverage	No	809(5.8)	81(8.1)	890(5.9)	9.083	0.002
	Yes	13,238(94.2)	921(91.9)	14,159(94.1)		
History of Heart Attack	No	13,704(97.6)	940(93.8)	14,644(97.3)	50.112	0.000
	Yes	343(2.4)	62(6.2)	405(2.7)		
Experience of Diabetes	No	12,234(87.1)	731(73.0)	12,965(86.2)	156.726	0.000
	Yes	1,813(12.9)	271(27.0)	2,084(13.8)		

### 3.2 Model estimation

**Table 2** displays the outcomes of the calculated binary logistic regression model. The study investigated the possibility of multicollinearity between the independent variables that were part of the model. Multicollinearity is considered moderate when the variance inflation factor (VIF) value is five or higher, and high when it is 10 or higher (98). There isn’t a single variable in this study that contributes to multicollinearity.

**Table 2:** The results of the binary logit model and marginal effects.

Variables	β	Std. error	Marginal effects	Std. error	VIF
Gender (reference: male)					
Female	0.771^a^	0.085	0.722^a^	0.080	1.51
Age (reference:65+)					
15-24	0.606^b^	0.3	0.563^b^	0.274	1.29
25-34	0.318^b^	0.151	0.298^b^	0.141	2.66
35-44	0.314^b^	0.127	0.295^b^	0.119	2.94
45-54	0.332^a^	0.118	0.311^a^	0.111	2.49
55-64	0.262^b^	0.113	0.247^b^	0.107	1.96
Educational Status (reference: Illiterate)					
Primary school	0.298^a^	0.112	0.280^a^	0.106	3.16
Elementary school	0.468^a^	0.143	0.439^a^	0.134	2.27
High school	0.478^a^	0.135	0.448^a^	0.127	2.72
University	0.368^b^	0.147	0.346^b^	0.138	2.88
Employment (reference: unemployed)					
Employed	-0.163^c^	0.091	-0.153^c^	0.085	1,69
General Health Status (reference: poor/very poor)					
Very good/good	-1.236^a^	0.11	-1.142^a^	0.101	4.45
Moderate	-0.665^a^	0.091	-0.602^a^	0.082	3.54
Experiencing Illness due to Substance Use (reference: no)					
Yes	0.890	1247	0.803	1.078	1.0
Disease Status (reference: no)					
Yes	3.300^a^	0.245	3.202^a^	0.243	1.6
Alcohol Use Status (reference: no)					
Yes	0.390^a^	0.115	0.360^a^	0.105	1.12
Tobacco Use Status (reference: no)					
Yes	0.490^a^	0.077	0.455^a^	0.071	1.17
E-cigarette Use Status (reference: no)					
Yes	-0.596	0.399	-0.565	0.383	1.01
SSI Coverage (reference: no)					
Yes	-0.344^a^	0.131	-0.318^a^	0.119	1.03
History of Heart Attack (reference: no)					
Yes	0.381^b^	0.149	0.351^b^	0.136	1.05
Experience of Diabetes (reference: no)					
Yes	0.175^b^	0.082	0.162^b^	0.077	1.22

^a^ p ^<^0.01; ^b^p ^<^0.05; ^c^p ^<^0.10.

Looking at **Table 2**, it is observed that in the established model, the variables gender, age, educational status, general health status, employment status, presence of chronic illness, alcohol use, tobacco use, Social Security Institution (SSI) coverage, history of heart attack, and history of diabetes are statistically significant.

The marginal effects of the variables associated with the probability of experiencing depression among married individuals are presented in **Table 2**. According to **Table 2**, the probability of experiencing depression among women is 72.2% higher than that of men. An individual in the 15–24 age group has a 56.3% higher probability of experiencing depression compared to an individual aged 65 and above (reference group). Similarly, individuals aged 25–34 have a 29.8% higher probability of experiencing depression compared to the reference group. In addition, the probability of experiencing depression is 29.5% higher for those aged 35–44, 31.1% higher for those aged 45–54, and 24.7% higher for those aged 55–64 compared to the 65 +.

**Table 2** shows that individuals with a primary school education have a 28% higher probability of experiencing depression compared to illiterate individuals (reference group). Those with an elementary school education are 43.9% more likely, high school graduates are 44.8% more likely, and university graduates are 34.6% more likely to experience depression than the reference group. Employed individuals have a 15.3% lower probability of experiencing depression compared to those who are unemployed. Individuals with very good/good general health status are 114.2% less likely to experience depression compared to those with poor/very poor general health status (reference group). Individuals with moderate health are 60.2% less likely to experience depression compared to the reference group.

In **Table 2**, individuals who have or are expected to have a disease/health problem lasting 6 months or longer are 320.2% more likely to experience depression than those without such a condition. The probability of experiencing depression among tobacco users is 45.5% higher than among non-users. Alcohol consumers are 36% more likely to experience depression than non-consumers. Individuals with Social Security Institution (SSI) coverage are 31.8% less likely to experience depression than those without SSI coverage. Individuals who had a heart attack in the past 12 months are 35.1% more likely to experience depression than those who did not. Similarly, individuals who were diagnosed with diabetes in the past 12 months are 16.2% more likely to experience depression than those who were not.

## 4 Discussion

This study provides one of the first large-scale, nationally representative analyses of depression among married individuals in Türkiye, offering important insights into how demographic, socioeconomic, and health-related factors interact within the context of marriage. Using binary logistic regression analysis, factors associated with the likelihood of experiencing depression within the last 12 months were identified among married individuals in Türkiye. The results indicated that gender, age, educational status, general health status, employment status, presence of chronic illness, alcohol use, tobacco use, Social Security Institution (SSI) coverage, history of heart attack, and history of diabetes were significantly associated with depression among married individuals.

Married women were found to have a higher probability of experiencing depression compared to married men. Consistent with this finding, previous studies conducted in various countries have shown that women tend to have higher levels of depression than men (73, 74, 99, 100). A previous study attributed this finding to differences in socioeconomic factors such as education, income, culture, diet, and harassment, which may contribute to higher rates of depression among women (71). Another study, however, suggested that biological sex differences and changes in ovarian hormones may contribute to the increased prevalence of depression among women (101). In contrast, a study conducted in Iran found that depression levels were higher among men than women (72). Although the empirical literature documents a range of variables related to depression, no comprehensive theory has been able to fully explain the gender differences reflected in the data (102). Current reductionist explanations tend to point to single causal factors, such as female hormones, thereby overlooking women’s complex experiences. Given that single explanatory theories cannot fully account for why depression rates are higher among women, the use of a social-structural theory can address broader questions regarding this gender difference while also avoiding the pitfalls of reductionism (103). A comprehensive examination of mental illnesses that affect genders unequally can contribute to understanding the social stresses and demands applied unequally to women and men (104). The general theory of gender stratification and the theory of gender and power are two social-structural theories that seek to explain social phenomena in terms of different power structures (105, 106). Biological sex takes on meaning in the social structure through specific gender roles defined by a male-dominated social system that excludes women from equal access to power. Although neither theory has been used in empirical studies on mental health issues, both contain micro-level components that could be useful in examining gender differences in depression, particularly among cohabiting women. The integration of concepts from these theories can provide a valuable tool for examining both conceptual issues and empirical data (107).

The study found that the likelihood of experiencing depression decreased with increasing age among married individuals. Similar findings have been reported in earlier studies (75, 76, 108, 109). One previous study attributed this to the observation that middle-aged and elderly couples with stronger emotional bonds with their children tend to have higher life satisfaction and lower levels of depression (75). Contrary to this finding in our study, the Life Course Theory suggests that a U-shaped, nonlinear curve typically represents the relationship between age and depressive symptoms; this curve drops sharply during early adulthood, reaches its lowest point in middle age, and then rises again around age 70 as role transitions such as retirement and widowhood, functional decline, and a diminished sense of control create challenges for mental health (110, 111).

According to the study, the likelihood of experiencing depression increased with higher levels of educational status among married individuals. According to some earlier research, the prevalence of depression varies according to a person’s educational attainment (112, 113). On the other hand, a different study indicated that depression risk was correlated with lower educational status (77). Previous studies in the literature have also found a negative association between educational level and depression (114–116). Research on the negative relationship between educational level and depression has followed two theoretical approaches: the school dropout theory and the protection theory. The school dropout theory (also known as “selection” in some literature) argues that children with depression have lower academic proficiency and a higher likelihood of dropping out of school, which ultimately leads to a lower educational level (117). Studies based on this theory may or may not claim causality, but they share the common thread of treating depression as an independent variable and emphasizing its negative impact on educational attainment (115, 116). The protection theory (known as “causality” in some literature) argues that a higher level of education provides protective benefits against depression and that, consequently, individuals with higher levels of education have lower levels of depression (117). It treats educational level as an independent variable and emphasizes its protective effect against depression. Many studies have explicitly or implicitly supported the protection theory (78, 118).

The study found that employed married individuals were less likely to experience depression compared to their unemployed counterparts. A previous study linked this to income level being an important factor in whether family members experience depression (52). Similarly, prior research has shown that income level is a significant variable influencing levels of depression (119–121).

The study also found that as the general health status of married individuals improved, their likelihood of experiencing depression decreased. According to a prior study with a Turkish sample, depressive symptoms were strongly predicted by one’s health situation (80). This finding is consistent with the predictions of the Stress Process Model. The model identifies poor health as a significant source of stress for individuals and posits that such stressors increase the risk of depression (122).

Additionally, compared to those without such diseases, married people who had a health issue or illness that lasted (or was predicted to last) six months or more were more likely to suffer from depression. Previous investigations have found similar findings (81, 123). Another earlier study conducted with elderly individuals suffering from chronic illnesses found high levels of depression among the elderly (124). The research indicated that individuals who are married and consume alcohol face an increased likelihood of experiencing depression compared to those who abstain from drinking. This situation can be explained within the framework of the Self-Medication Theory as follows: individuals who use alcohol to alleviate depressive feelings may actually increase their risk of depression in the long term (125). A previous study found that 25% of individuals with depression use alcohol to alleviate their symptoms (85). According to earlier studies, substance abuse and alcohol consumption are linked to depressive symptoms and may be a contributing factor or a coping strategy for self-medication (16, 126). Furthermore, a previous study has highlighted that linking mood disorders to alcohol dependence may increase the risk of suicide (127).

Additionally, married tobacco users are more likely to suffer from depression than non-users, according to the study. A prior study found a correlation between tobacco use and seeking mental health treatment within the previous 12 months (91). According to a previous study conducted in a Turkish sample, anxiety and depression, along with factors such as feelings of loneliness and a lack of love, are cited as reasons why young people start smoking (128).

Research has shown that individuals who are married and receive benefits from the Social Security Institution (SSI) tend to experience lower rates of depression compared to their unmarried counterparts. This is supported by a prior study that discovered a significant correlation between marital financial uncertainty and depressive symptoms (16).

Married individuals who experienced a heart attack within the past 12 months were more likely to experience depression than those who did not. This situation can be explained within the framework of the Stress Process Model as follows: serious physical health problems can cause stress in an individual, thereby increasing the likelihood of developing depression (122). A previous study using a Turkish sample found that patients with heart conditions exhibited significantly higher levels of depression compared to those with other chronic illnesses (81). A systematic review also highlighted that depression is significantly more prevalent among individuals with cardiovascular disease and is associated with higher mortality and morbidity rates (84).

According to the study, married individuals who experienced diabetes within the past 12 months had a higher likelihood of experiencing depression compared to those who did not. This situation can be explained within the framework of the Stress Process Model as follows: serious physical health problems can act as a source of stress, thereby increasing the risk of depression (122). A previous study indicated that during depressive episodes, patients often experience an increase in physical problems such as irregular blood pressure and blood glucose levels (81).

From a theoretical perspective, the findings contribute to the ongoing debate between the marital protection and marital selection hypotheses. The results suggest that both mechanisms operate simultaneously in the Turkish context. On the one hand, the lower likelihood of depression among individuals with better socioeconomic and health conditions supports the selection hypothesis, indicating that healthier and more advantaged individuals are less likely to experience depression within marriage. On the other hand, the significant role of employment, social security coverage, and health status highlights the protective function of structural and institutional factors, consistent with the marital protection hypothesis.

Therefore, this study extends the literature by demonstrating that the relationship between marriage and depression should be understood within a broader socioeconomic and institutional framework rather than as a purely individual-level phenomenon.

## 5 Conclusion

Marriage and family relationships are among the most significant and essential social structures in every society. One of the most important social environments for children’s, teens’, and adults’ health is the family. Therefore, improving the family environment will be beneficial not only for individuals but also for society. Social policies are needed to improve the family environment.

The study findings indicate that the risk of depression among married individuals varies depending on gender, age, education, employment status, general health, alcohol and tobacco use, and social security coverage. Accordingly, it is important to expand mental health screening programs and psychosocial support services for the prevention and early intervention of depression, conduct awareness campaigns to reduce alcohol and tobacco use, and strengthen social security and economic support mechanisms. The design of these recommendations should take into account the distribution of existing medical resources and Türkiye’s social policy framework, thereby enhancing their feasibility and effectiveness.

In terms of policy implications, the findings highlight the need for targeted and context-specific interventions in Türkiye. Given the higher risk of depression among individuals with chronic illnesses and poorer health status, integrating mental health screening into primary healthcare services could be an effective strategy. In addition, expanding community-based mental health services and ensuring equitable access across regions would improve early detection and intervention.

Furthermore, given the protective role of employment and social security coverage, policies aimed at strengthening labor market participation and expanding social protection mechanisms may help reduce the risk of depression. Tailored interventions targeting younger married individuals and women—who are identified as high-risk groups—should also be prioritized. These measures should align with Türkiye’s existing healthcare infrastructure and social policy framework to ensure feasibility and sustainability.

Importantly, these policy recommendations should be implemented by considering regional disparities in healthcare access and socioeconomic inequalities across Türkiye. Integrating mental health services into primary healthcare, particularly for individuals with chronic illnesses, and expanding community-based psychosocial support systems may enhance early detection and intervention. In addition, targeted programs focusing on young married individuals and women—identified as higher-risk groups in this study—should be prioritized to improve effectiveness and policy relevance.

There are some drawbacks to this study. It uses secondary data to start. Only variables already included in the dataset are needed for statistical analysis. Second, a clear causal link between the depression status of married people and the related socioeconomic determinants cannot be demonstrated because the data are cross-sectional. Thirdly, since no laboratory-based clinical tests were conducted to assess the depression status of married individuals, the findings of the study are based on the participants’ self-reports. Therefore, it can be said that the data gathered with this data collection technique might be subject to bias.

Although this study provides important insights into the factors associated with depression among married individuals, potential endogeneity issues should be acknowledged. First, reverse causality may exist between some explanatory variables and depression. For example, while unemployment may increase the likelihood of depression, depression itself may reduce individuals’ capacity to participate in the labor market. Similarly, health conditions may both influence and be influenced by depressive symptoms. Second, omitted-variable bias may arise from unobserved factors, such as personality traits, social support networks, or marital quality, that are not available in the dataset but may affect both the independent variables and the depression outcomes.

Moreover, given the cross-sectional nature of the data, causal interpretations cannot be established. The results should therefore be interpreted as associations rather than causal effects. Future studies using longitudinal data or quasi-experimental designs are needed to address endogeneity concerns better and establish causal relationships.

Another important limitation concerns the measurement of depression. The dependent variable is based on a single self-reported item indicating whether the individual experienced depression within the past 12 months. This measure does not correspond to a clinical diagnosis nor to a validated multi-item scale such as the PHQ-9 or BDI. Self-reported measures are subject to reporting bias, including underreporting due to social stigma or differences in individual perception of mental health.

Although previous studies have shown that single-item mental health indicators can serve as useful proxies in large-scale epidemiological research, the results should be interpreted with caution. Future studies are encouraged to incorporate clinically validated instruments to improve measurement accuracy.

## Data Availability

The data underlying this study is subject to third-party restrictions by the Turkish Statistical Institute. Data are available from the Turkish Statistical Institute (bilgi@tuik.gov.tr) for researchers who meet the criteria for access to confidential data. Further inquiries can be directed to the corresponding author/s.
